# Fexuprazan and Esomeprazole in Patients with Disorders Associated with Acid Reflux: A Comprehensive Review and Meta-Analysis of Randomized Controlled Trials

**DOI:** 10.3390/jcm15041434

**Published:** 2026-02-12

**Authors:** William A. Barzola-Farfán, Carlos Quispe-Vicuña, Oriana Rivera-Lozada, Cesar Bonilla-Asalde, Joshuan J. Barboza

**Affiliations:** 1 Instituto de Evaluación de Tecnologías en Salud e Investigación, EsSalud, Lima 15072, Peru; william.barzola@upch.pe; 2 Tau Clinical Research Methods Network, Trujillo 13001, Peru; vicunas998@gmail.com; 3 Vicerrectorado de Investigación, Universidad Señor de Sipan, Chiclayo 14001, Peru; riveraoriana@uss.edu.pe (O.R.-L.); bonasal@gmail.com (C.B.-A.)

**Keywords:** fexuprazan, DWP14012, esomeprazole, erosive esophagitis, laryngopharyngeal reflux disease

## Abstract

**Background**: This investigation compared the efficacy and safety of fexuprazan 40 mg and esomeprazole 40 mg in patients with acid reflux-related disorders, including erosive esophagitis (EE) and laryngopharyngeal reflux disease (LPRD). **Methods**: A systematic search was conducted across five databases until January 2025. Primary outcomes included esophageal lesion healing, complete resolution of symptoms (CRS), and 24 h symptom-free days. Meta-analyses used random-effects models with the inverse variance method. The Risk of Bias 2.0 tool and the certainty of evidence (CoE) using GRADE methodology were assessed. **Results**: Three randomized controlled trials (n = 695) conducted in Asian countries were included. Fexuprazan may have little to no effect compared to esomeprazole on EE healing rate at 4 weeks (RR 1.02, 95% CI 0.93 to 1.12, I^2^ = 0%, n = 2 studies, CoE very low) and 8 weeks. No significant differences were found between treatments regarding CRS at 1 week (RR 1.29; 95% CI: 0.84 to 1.99; I^2^ = 0%; n = 2 studies; CoE very low) and 8 weeks, or in the 24 h symptom-free days at 1 week (MD 2.67 days, 95% CI −2.76 to 8.10, I^2^ = 41%, n = 2 studies, CoE very low) and 8 weeks. Fexuprazan also showed little to no effect on treatment-emergent adverse events (RR 1.00, 95% CI 0.83 to 1.20, I^2^ = 0%, n = 3 studies, CoE very low). Nonetheless, the evidence for all outcomes was rated as very uncertain. **Conclusions**: Fexuprazan 40 mg may provide similar efficacy compared to esomeprazole 40 mg in EE, with a comparable safety profile to esomeprazole in EE and LPRD patients. However, the evidence is highly uncertain, requiring further studies.

## 1. Introduction

Gastroesophageal reflux disease (GERD) and laryngopharyngeal reflux disease (LPRD) are classified as acid reflux-related disorders (ARDs), which are characterized by excessive gastric acid secretion or increased sensitivity to gastric contents [1].

GERD affects approximately 14% of the global population and results from the reflux of stomach contents into the esophagus [2,3], which may lead to erosive esophagitis (EE), a condition characterized by damage to the esophageal mucosa [4]. EE has an estimated pooled frequency of 28% among individuals with GERD and commonly presents with symptoms such as dysphagia, heartburn, and sore throat [5]. Furthermore, LPRD, an extra-esophageal manifestation of reflux illness, has been reported with a prevalence ranging from 7.1% to 30% [6]. LRPD results from the retrograde migration of stomach contents into the larynx and pharynx, which manifests as symptoms such as dysphagia, sore throat, and dysphonia [7]. LPRD has been linked to chronic conditions such as sinusitis, otitis media, asthma, and laryngeal cancer, while untreated GERD may progress to complications such as Barrett’s esophagus or esophageal adenocarcinoma [8,9].

Reducing stomach acid exposure is a major therapeutic goal aimed to lessen mucosal injury, particularly given the significant clinical burden associated with ARDs [8]. Therefore, acid-suppressing drugs like proton pump inhibitors (PPIs) are the first-line treatment for both EE and LPRD [8,9]. Despite their excellent healing rates, about 5% to 30% of patients with EE fail to completely resolve their lesions or symptoms after eight weeks of therapy [2,10]. Furthermore, genetic polymorphisms in the CYP2C19 enzyme, which are responsible for PPI metabolism, may contribute to interindividual variability in drug exposure, which may result in differences in both efficacy and adverse effects [11].

Potassium-competitive acid blockers (P-CABs) represent a promising therapeutic alternative, as they constitute a new class of acid-suppressing drugs [12]. These compounds suppress the production of gastric acid by inhibiting the gastric H^+^, K^+^-ATPase enzyme in a reversible and competitive manner [13]. Compared to PPIs, fexuprazan, a recently approved P-CAB in some Asian countries, has demonstrated unique pharmacologic and pharmacokinetic properties, such as a faster onset of action and a longer duration of acid suppression [14].

A growing number of randomized controlled trials (RCTs) have evaluated the acid-suppressive properties and safety profiles of fexuprazan in comparison to esomeprazole, a commonly used PPI. In an 8-week trial, Lee et al. reported similar healing rates for EE between fexuprazan and esomeprazole [15]. Similarly, an RCT conducted by Zhuang et al. found no significant differences among the two agents in the cure rates of EE at weeks 4 and 8, symptom improvement, and changes in gastroesophageal reflux disease health-related quality of life (GERD-HRQL) scores [16]. Furthermore, Kim et al. found no discernible variations in adverse drug responses (ADRs) or treatment-emergent adverse events (TEAEs) among patients with LPRD with either drug [17]. Despite these findings, there is still uncertainty regarding the advantages and disadvantages of fexuprazan compared to esomeprazole, one of the most commonly prescribed PPIs for EE and LPRD. Although a previous network meta-analysis by Wang et al. [1] evaluated the efficacy of P-CABs, including fexuprazan, recent RCTs published after their search date provided new evidence that warranted the present study. Thus, the goal of this systematic study was to compare the safety and efficacy of fexuprazan and esomeprazole in patients with ARDs, and to assess the certainty of evidence.

## 2. Materials and Methods

### 2.1. Research Design

We conducted a systematic review and meta-analysis in accordance with the Preferred Reporting Items for Systematic Reviews and Meta-Analyses (PRISMA) 2020 criteria [18] (Table S1). We used the identification code CRD42025591424 to register a protocol in PROSPERO.

### 2.2. Searches

We performed an extensive literature search across PubMed, Scopus, the Cochrane Library, Web of Science, and EMBASE from their inception until January 2025. The search approach incorporated MeSH, Emtree words, and key phrases. A particular approach was used for each database, with the primary search terms being (“Esophagitis”) AND (“Fexuprazan”) AND (“Esomeprazole”). No restrictions on language or publication date were taken into consideration. Table S2 provides the whole search strategy. In addition, the reference lists of all pertinent papers and review articles were also carefully screened to find any possibly qualifying trials.

### 2.3. Criteria for Eligibility

Eligible studies included Phase II and Phase III RCTs involving patients with ARDs (EE or LPRD) who were 18 years of age or older. Studies that met the eligibility requirements evaluated fexuprazan (40 mg once daily) as an intervention in comparison to esomeprazole (40 mg once daily). Letters to the editor, case reports and series, narrative reviews, systematic reviews, and conference abstracts were not included. 

The main outcomes were the healing of esophageal lesions, which was verified by esophagogastroduodenoscopy (healing rate at weeks 1 and 8), and patient-reported symptom improvement, as indicated by 24 h symptom-free days at weeks 1 and 8 and complete symptom resolution (CSR). Secondary outcomes included changes in GERD-HRQL scores at weeks 4 and 8. In addition, we evaluated safety results according to the frequency of ADRs and TEAEs.

### 2.4. Data Extraction

Following the computerized searches, duplicates were removed and the remaining results were consolidated into a singular reference library. Two researchers (WABF and CVQ) independently evaluated the titles and abstracts during the initial screening phase, utilizing the Rayyan QCRI platform “https://rayyan.qcri.org/ (accessed on 31 January 2025)” to implement the inclusion and exclusion criteria [19]. A subsequent screening was performed to reinforce the inclusion and exclusion criteria for full-text assessment to studies that met eligibility requirements. A third review author (JJB) was consulted to resolve discrepancies.

To ensure a blinded and systematic methodology, two authors (WABF and CQV) independently collected data from each study utilizing a pre-designed Excel spreadsheet for data extraction. The author, year of publication, country, study type, participant count per intervention arm, selection criteria, descriptions of interventions and controls, as well as primary and secondary outcomes for each study were collected.

### 2.5. Assessment of the Risk of Bias

Two authors (WABF and CQV) independently assessed the risk of bias (RoB) utilizing the RoB 2.0 methodology [20]. This tool examines various domains where bias may emerge, including the randomization process, deviations from intended interventions (effects of intervention allocation), absent outcome data, outcome evaluation, and selection of reported results. Consultations with a third author (JJB) facilitated the resolution of disagreements. The risk of bias for RCTs was classified as low, some concerns, or high for each domain and study.

### 2.6. Synthesis of Data

All meta-analyses assessing the effects of fexuprazan and esomeprazole were conducted using random-effects models with the inverse variance method. The tau^2^ variance between studies was calculated employing the Paule-Mandel method. Continuous outcomes were presented as mean differences (MD) with standard deviations (SD), while the effects of fexuprazan 40 mg compared to esomeprazole 40 mg on dichotomous outcomes were expressed as relative risks (RR) with 95% confidence intervals (CI). The continuity correction method was employed to adjust for zero occurrences in one or both arms of the RCTs. When the meta-analysis included more than five studies, the Hartung-Knapp method was used. The I^2^ statistic was employed to assess statistical heterogeneity among RCTs, with values interpreted as low (<30%), moderate (30–60%), and high (>60%) heterogeneity. The Mantel–Haenszel technique and fixed effects were utilized for sensitivity analysis. We utilized the matacont and metabin functions from the R 4.3.3 meta package (www.r-project.org). Egger’s test and funnel plots were employed to evaluate publication bias in studies with a minimum of ten articles.

### 2.7. GRADE Evaluation

The certainty of evidence (CoE) for both dichotomous [21] and continuous [22] outcomes were assessed utilizing the GRADE methodology. This assessment analyzed five domains, including publication bias, indirectness, imprecision, inconsistency, and risk of bias. The CoE for each outcome was assessed and detailed in the Summary of Findings (SoF) tables, generated using the online software GRADEpro GDT. Copyright© 2021, Evidence Prime Inc., Hamilton, ON, Canada, and McMaster University. Moreover, the authors declare that generative artificial intelligence (GenAI) was not used in this study.

## 3. Results

### 3.1. Study Selection

**Figure 1:**
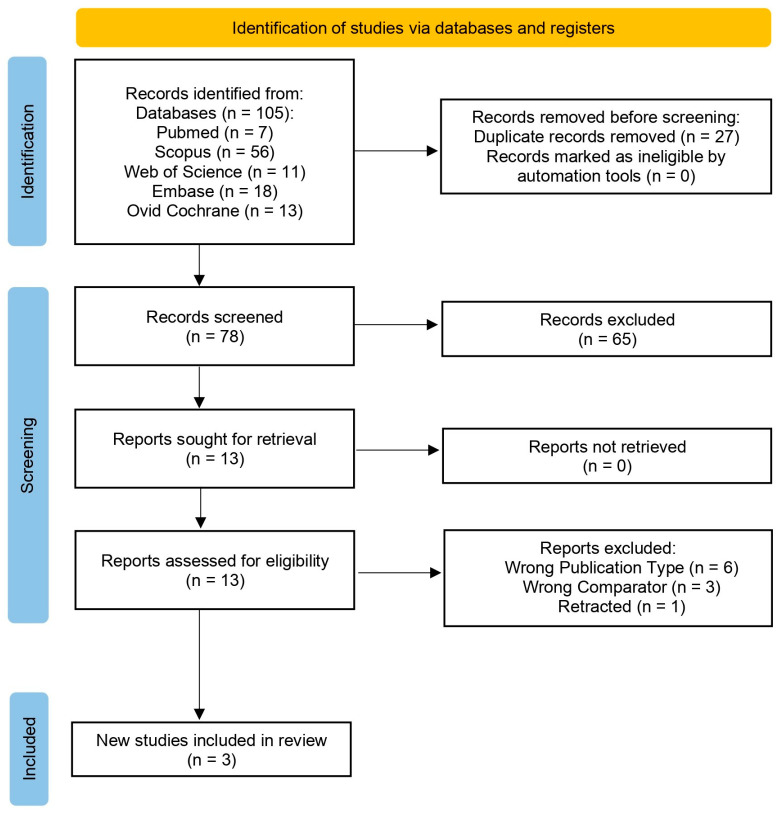
PRISMA 2020 flowchart of the study selection process.

Of the 105 studies identified in various databases, 27 duplicates were eliminated. Lastly, the systematic review included three trials [15,16,17] (Figure 1). Table S3 lists the omitted articles along with the explanations for their exclusion.

### 3.2. Features of the Included Research

The included studies have been fully described, including design, population, interventions, comparators, and outcomes. All three were a double-blind phase III RCTs carried either in China or South Korea. A total of 695 patients were evaluated (esomeprazole = 346; fexuprazan = 349). Most patients in the fexuprazan group (69.99%) were male, and the mean age was 51.09 years (SD: 12.84). Similarly, the majority of individuals (67.52%) in the esomeprazole group were male, and the average age was 51.22 years (SD: 13.61) (Table 1).

All included studies were RCTs that assessed the safety and efficacy of fexuprazan versus esomeprazole for the treatment of ARDs. While the study by Kim et al. [17] focused on patients with LPRD, the studies by Lee et al. [15] and Zhuang et al. [16] assessed patients with EE. In these trials, fexuprazan was used as the intervention at a dose of 40 mg taken orally once daily for a maximum of 8 weeks, in addition to a placebo. The control group received 40 mg of esomeprazole following the same dosage and schedule, along with a placebo. Although reported outcomes varied across studies, they commonly included clinical measures such as symptom relief and mucosal healing rates, as well as quality of life ratings (e.g., GERD-HRQL score).

The frequency of side effects, such as headache, nausea, diarrhea, dizziness, and abdominal pain, were secondary outcomes. In order to ascertain which medication offered superior symptom relief, safety, and quality of life in patients with EE and LPRD, these trials compared fexuprazan and esomeprazole. No serious adverse effects were reported in the fexuprazan groups in any of the investigations (Table 2).

### 3.3. Assessment of the Risk of Bias

**Figure 2:**
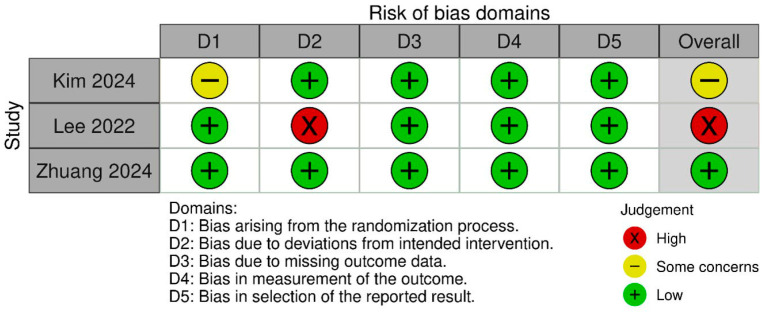
Risk of bias assessment of included studies according to Cochrane risk-of-bias tool for randomized trials (RoB 2.0) [15,16,17].

Due to a paucity of information describing the blinding procedure, the study by Kim et al. [17] raised some concerns regarding the risk of bias. On the other hand, due to methodological flaws in the participant analysis, the study by Lee et al. [15] exhibited a high risk of bias (Figure 2).

### 3.4. GRADE of Evidence Assessment Certainty

A very low CoE was reported for all outcomes. Since all included studies were RCTs, the assessment started with high certainty. However, the width of the CI, the absence of statistical significance for all outcomes, and the high and moderate risk of bias found in two studies downgraded the CoE (Table 3).

#### Effects of Fexuprazan 40 mg on the Primary and Secondary Results

**Figure 3:**
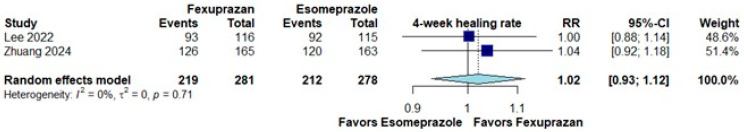
Forest plot of pooled 4-week healing rate [15,16].

**Figure 4:**
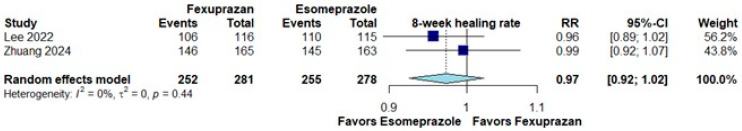
Forest plot of pooled 8-week healing rate [15,16].

**Figure 5:**
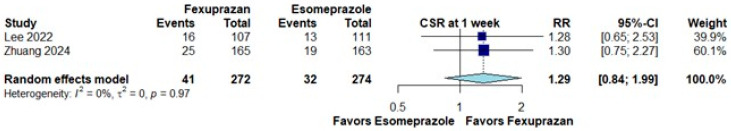
Forest plot of pooled estimates for complete symptom resolution (CSR) at 1 week [15,16].

Fexuprazan 40 mg may have little to no effect on the 4-week healing rate of EE compared to esomeprazole 40 mg, but the evidence is very uncertain (RR 1.02, 95% CI 0.93 to 1.12, I^2^ = 0%, n = 2 studies, CoE very low) (Figure 3). Similarly, the effect fexuprazan 40 mg may not differ from esomeprazole 40 mg regarding the healing rate after 8 weeks, however the confidence in the effect estimate is very limited due to uncertainty (RR 0.97, 95% CI 0.92 to 1.02, I^2^ = 0%, n = 2 studies, CoE very low) (Figure 4). Moreover, no significant differences were observed between fexuprazan 40 mg and esomeprazole 40 mg regarding CSR at 1 week (RR 1.29, 95% CI 0.84 to 1.99, I^2^ = 0%, n = 2 studies, CoE very low) (Figure 5) or at 8 weeks (RR 1.10, 95% CI 0.89 to 1.35, I^2^ = 0%, n = 2 studies, CoE very low) (Figure S1). Nevertheless, the reliability of the estimates is restricted due to relevant uncertainty. Fexuprazan 40 mg may result in minimal to no effect in comparison with esomeprazole 40 mg on the 24 h symptom-free days at 1 week (MD 2.67 days, 95% CI −2.76 to 8.10, I^2^ = 41%, n = 2 studies, CoE very low) (Figure S2) and at 8 weeks of treatment (MD 0.08 days, 95% CI −6.52 to 6.67, I^2^ = 56%, n = 2 studies, CoE very low) (Figure S3). However, the trustworthiness of these estimates is limited due to the high level of uncertainty. No differences were observed in the effect of fexuprazan 40 mg compared to esomeprazole 40 mg on changes in quality of life as determined by the GERD-HRQL score at 4 weeks (MD 0.48, 95% CI −0.21 to 1.17, I^2^ = 0%, n = 2 studies, CoE very low) (Figure S4) or at 8 weeks (MD 0.37, 95% CI −0.31 to 1.05, I^2^ = 0%, n = 2 studies, CoE very low) (Figure S5). Nonetheless, confidence in the effect estimates is very limited given substantial uncertainty in evidence.

In terms of safety outcomes, fexuprazan 40 mg may have minimal to no effect compared to esomeprazole 40 mg on the occurrence of TEAEs (RR 1.00, 95% CI 0.83 to 1.20, I^2^ = 0%, n = 3 studies, CoE very low) (Figure S6) and ADRs (RR 1.05, 95% CI 0.72 to 1.54, I^2^ = 0%, n = 3 trials, CoE very low) (Figure S7). Furthermore, no significant differences were found among fexuprazan 40 mg and esomeprazole 40 mg in the incidence of specific adverse effects, including headache, abdominal pain, dizziness, nausea and diarrhea (Figures S8–S12). However, similar to the findings for TEAEs and ADRs, the evidence is highly uncertain.

## 4. Discussion

According to this meta-analysis, fexuprazan 40 mg may have comparable efficacy and safety to esomeprazole 40 mg in adult patients with ARDs. In particular, fexuprazan appears to have little to no additional effect in comparison with esomeprazole on improving symptom resolution, GERD-HRQL and mucosal healing rate of EE at 4 and 8 weeks. Similarly, no significant differences were observed in the incidence of TEAEs or ADRs in patients with EE or LPRD. These results imply that fexuprazan may offer clinical advantages comparable to those of esomeprazole in the treatment of ARDs in adult patients. However, given the very low certainty of evidence, these results are of poor reliability and should be interpreted cautiously, as future research may differ from existing estimations.

In comparison with esomeprazole 40 mg, fexuprazan 40 mg may result in a similar healing rate for EE at 4 and 8 weeks. This finding is consistent with the results of a network meta-analysis conducted by Wang et al. [1], which, based on a single study included in this systematic review [15], reported no significant differences in the odds of mucosal healing in EE between esomeprazole 40 mg and fexuprazan 40 mg (OR 1.04, 95% CI 0.06 to 16.80). Moreover, our findings align with those of Lee et al. [15], which found that at 4 weeks, the healing rate for EE with fexuprazan 40 mg was 2.6% higher than with esomeprazole 40 mg, although the confidence interval included the possibility of no difference (95% CI −5.7 to 10.9). Similarly, Zhuang et al. [16] reported a non-significant difference of −0.4% (95% CI −7.2 to 6.5) in the healing rate at 8 weeks between both drugs in adults with EE. In addition to its acid-suppressive activity, fexuprazan may also possess anti-inflammatory properties. In an in vitro study by Kim et al. [23], fexuprazan showed protective effects on human esophageal cells against hydrochloric acid-induced injury by suppressing the expression of genes involved in the pyroptosis pathway in esophagitis, such as NOD-like receptor family pyrin domain containing 1 (NLRP1), caspase-1, gasdermin D, and IL-1β. Furthermore, in clinical research that included patients with acute and chronic gastritis, fexuprazan 20 mg was more effective than a placebo in terms of stomach erosion healing rates (n = 59/102; 57.8% vs. n = 39/96; 40.6%; *p* = 0.017) [12]. These results suggest that, although fexuprazan may exert effects similar to those of esomeprazole, it may enhance the recovery of gastric acid-induced mucosal tissue damage through more than only one mechanism.

Additionally, we found that fexuprazan 40 mg, compared to esomeprazole 40 mg, may have little to no effect on the management of symptoms in individuals with esophageal lesions at both 1 and 8 weeks. This result aligns with the RCT by Zhuang et al. [16], which found a non-significant difference of 1.5% (95% CI −9.9 to 12.9) in the rate of complete resolution at 8 weeks between both drugs. Despite the well-known advantages of P-CABs, including a quicker onset of action, increased stability in acidic environments without the requirement for an enteric coating, and a longer duration of acid suppression compared to PPIs [1,24], fexuprazan did not demonstrate additional benefits over esomeprazole in this systematic review. However, it is relevant to consider that this drug does not necessitate dose modifications based on genetic variants that affect individual variability given its metabolism is unaffected by CYP2C19 enzymes [13]. Thus, for adults with ARDs who may need both acid suppression and anti-inflammatory effects, these special pharmacokinetic and pharmacodynamic qualities—which are still poorly understood—may offer particular benefits, which should be further investigated in future studies.

In terms of safety outcomes, fexuprazan 40 mg showed to have minimal to no effect on the incidence of TEAEs and ADRs compared to esomeprazole 40 mg. Our findings are compatible with those of Wang et al. [1], who reported no differences in the odds of total adverse events in patients with EE between esomeprazole and fexuprazan (OR 1.19, 95% CI 0.62 to 2.25). Similarly, the results align with the study by Kim et al. [17], that demonstrated no significant differences in the incidence of ADRs (n = 5/75; 6.67% vs. n = 4/75; 5.33%; *p* = 1.000) or TEAEs (n = 16/75; 21.33% vs. n = 17/75; 22.67%; *p* = 1.000) compared to esomeprazole in patients with LPRD, as well as no serious adverse events were identified. Although not exerting superior safety outcomes compared to esomeprazole, fexuprazan presents pharmacological properties that may enhance patients’ tolerability. In an open-label study by Lee et al. [25], fexuprazan demonstrated consistent healing rates of EE regardless of whether it was administered before or after meals. Furthermore, unlike PPIs, CYP2C19 enzymes have no effect on the metabolism of fexuprazan, which may lower the likelihood of drug overexposure and associated negative side effects [11]. These characteristics might lead to better patient adherence and increased therapy tolerability, although this hypothesis requires further confirmation in future real-world studies.

When compared to esomeprazole, fexuprazan may offer similar clinical advantages in terms of healing rates and symptom relief in adult patients with EE, as current evidence does not support superiority. Despite the fact that efficacy outcomes did not achieve statistical significance, fexuprazan 40 mg seems to have a therapeutic profile that is not inferior to esomeprazole 40 mg. Additionally, its safety profile appears to be similar to that of esomeprazole, with no significant adverse events reported in patients with EE or LPRD. Nonetheless, these results should be interpreted with caution due to the very low level of certainty of the evidence. There are several limitations on this study. All included studies were carried out in Asian countries, including China and South Korea, which may limit the generalizability of the findings to other populations. The number of RCTs and the sample sizes may have been insufficient to identify meaningful differences between patients treated with fexuprazan and esomeprazole. Future research with larger sample sizes may be able to uncover these potential differences. The absence of subgroup analyses based on the severity of EE or LPRD may limit a comprehensive understanding of the impact of the intervention across various patient subpopulations. Furthermore, the evaluation of the long-term safety and efficacy of both drugs is constrained by the relatively short follow-up duration of up to eight weeks. Finally, the fact that all included studies were funded by a single pharmaceutical corporation raises concerns regarding the possible impact on the independence of the stated findings. Future research is therefore required to validate these results.

## 5. Conclusions

Fexuprazan 40 mg, compared to esomeprazole 40 mg, may offer similar efficacy in adult patients with EE, as current evidence does not support its superiority. Additionally, it appears to possess a comparable safety profile to esomeprazole 40 mg in patients with EE and LPRD. However, the evidence remains highly uncertain.

## Figures and Tables

**Table 1:** Characteristics of included studies (n = 3).

Author, Year	NCT Code	Type and Phase	Country, Recruitment Dates	Participants—n	Inclusion Criteria	Exclusion Criteria	Sex (Male), %	Age (Mean ± SD)	Funding
Lee et al., 2022 [15]	NCT03736369	Double-blind, parallel-group, multicenter, and phase III RCT	South Korea, December 2018 to August 2019	Fexuprazan: 116 Esomeprazole: 115	(1) Adults aged 20–75 years (2) Documented diagnosis of EGRD classified as LA Grade A-D on upper gastrointestinal endoscopy (3) Presence of heartburn or acid regurgitation within the previous 7 days	(1) Individuals with a history of gastric acid suppression or who have undergone gastric or esophageal surgery (2) Individuals with other clinically significant disorders	Fexuprazan: 67.2 Esomeprazole: 64.3	Fexuprazan: 53.7 ± 12.44 Esomeprazole: 55.05 ± 12.89	Daewoong Pharmaceutical Co., Ltd.
Zhuang et al., 2024 [16]	NCT05813561	Double-blind, parallel-group, multicenter, and phase III RCT	China, December 2021 to February 2023	Fexuprazan: 165 Esomeprazole: 163	(1) Adults aged 18–75 years (2) Confirmed diagnosis of reflux esophagitis (Grade A-D according to LA classification) by EGD within 7 days prior to randomization	(1) Individuals with a known allergy to study drugs or unable to undergo EGD (2) Individuals with other clinically significant disorders. (3) Individuals with concomitant diseases that may affect esophageal motility, or a history of esophageal radiotherapy or cryotherapy (4) Individuals who had undergone surgery affecting the structure or function of the esophagus, stomach, or duodenum (5) Individuals requiring continuous treatment during the study period (6) Pregnant or lactating women	Fexuprazan: 79.4 Esomeprazole: 80.4	Fexuprazan: 49.2 ± 12.3 Esomeprazole: 46.9 ± 13.2	Daewoong Pharmaceutical Co., Ltd.
Kim et al., 2024 [17]	ISRCTN, KCT0007251	Double-blind, parallel-group, multicenter, and phase III RCT	South Korea, August 2022 to December 2023	Fexuprazan: 68 Esomeprazole: 68	(1) Adults aged ≥ 19 years (2) Presence of LPRD symptoms ≥ 1 month (3) RSI ≥ 13 (4) RFS ≥ 7	(1) Individuals with a history of head and neck, esophageal or gastric neoplasm (2) Prior radiotherapy or anti-reflux/gastro-esophageal surgery (3) Individuals with gastrointestinal disorders (erosive GERD, erosive esophagitis, Barrett’s esophagus, or Zollinger-Ellison syndrome) (4) Use of H2-blockers, PPIs, P-CABs, antacids, or prokinetics within prior two weeks (5) Abnormal laboratory results at screening or during follow-up	Fexuprazan: 51.9 Esomeprazole: 42.11	Fexuprazan: 51.2 ± 13.97 Esomeprazole: 55.1 ± 12.63	Daewoong Pharmaceutical Co., Ltd.

EGD: esophagogastroduodenoscopy; EGRD: Erosive gastroesophageal reflux disease; GERD: Gastroesophageal reflux disease; LA: Los Angeles Classification; LPRD: Laryngopharyngeal reflux disease; P-CAB: Potassium-competitive acid blocker; PPI: Proton pump inhibitor; RCT: randomized controlled trial; RFS: Reflux finding score; RSI: Reflux symptom index.

**Table 2:** Features of intervention and control of included studies (n = 3).

Author, Year	Intervention		Control	Outcomes
	Drug and Dosage	Description	Description	
Lee et al., 2022 [15]	Fexuprazan 40 mg, placebo-matching tablet, orally, once daily for up to 8 weeks plus Esomeprazole 40 mg placebo	Endoscopy was performed at baseline and at weeks 4 and 8. EE healing was defined as the complete absence of mucosal tears. If mucosal healing was not achieved by week 4, patients continued treatment until week 8, when endoscopy was repeated. Two weeks after confirmation of healing, patients were assessed for safety by telephone interviews.	Esomeprazole 40 mg placebo-matching tablet, orally, once daily for up to 8 weeks plus Fexuprazan 40 mg placebo	(1) Cumulative healing rate of EE at 4 and 8 weeks by EGD (2) Reflux disease symptom assessment using RDQ at 4 and 8 weeks (3) Quality of Life assessment using GERD-HRQL at 4 and 8 weeks (4) Adverse events
Zhuang et al., 2024 [16]	Fexuprazan 40 mg, orally, once daily (before meals) for up to 8 weeks plus Esomeprazole 40 mg placebo	After the initial visit, a repeat EGD was performed at 4 and 8 weeks. If healing was observed at week 4 (absence of mucosal tears according to LA classification), treatment was discontinued. Otherwise, the subject would receive another 4 weeks of treatment. Two weeks after the end of the treatment phase, patients were assessed for safety by telephone interviews.	Esomeprazole 40 mg, orally, once daily (before meals) for up to 8 weeks plus Fexuprazan 40 mg placebo	(1) Healing rate of EE at 4 and 8 weeks (2) Complete resolution of symptoms in the first 7 days, 4 weeks and 8 weeks. (3) Symptom-free days in the first 7 days, 4 weeks and 8 weeks. (4) Severity of predominant symptoms and quality of life (GERD-HRQL) at 4 and 8 weeks. (5) Adverse events
Kim et al., 2024 [17]	Fexuprazan 40 mg, orally, once daily (before breakfast) for 8 weeks plus Esomeprazole 40 mg placebo	At the first visit, baseline clinical measurements were performed, and 2 weeks later, quality of life was also assessed. These assessments were then repeated at 4 and 8 weeks, respectively.	Esomeprazole 40 mg, orally, once daily (before breakfast) for 8 weeks plus Fexuprazan 40 mg placebo	(1) Mean change in RSI score at week 4 and 8. (2) Rate of change and validity of RSI and RFS scores at weeks 4 and 8. (3) Mean change and validity of RSS-12 total and QoL scores at weeks 4 and 8. (4) Mean change in LPR-HRQoL scores at weeks 4 and 8.

EE: Erosive Esophagitis; RDQ: Reflux disease questionnaire; GERD-HRQL: GERD-Health related quality life; EGD: esophagogastroduodenoscopy; RSI: Reflux symptom index; RFS: Reflux finding score; QoL: quality of life; LPR-HRQoL: Laryngopharyngeal reflux–health-related quality of Life.

**Table 3:** Summary of finding (SoF).

Outcomes	Anticipated Absolute Effects * (95% CI)		Relative Effect (95% CI)	No. of Participants (Studies)	Certainty of the Evidence (GRADE)	Comments
	Risk with Esomeprazole 40 mg	Risk with Fexuprazan 40 mg				
4-week healing rate	763 per 1000	778 per 1000 (709 to 854)	RR 1.02 (0.93 to 1.12)	559 (2 RCTs)	⨁◯◯◯ Very low ^a,b^	Population: Patients with EE
8-week healing rate	917 per 1000	890 per 1000 (844 to 936)	RR 0.97 (0.92 to 1.02)	559 (2 RCTs)	⨁◯◯◯ Very low ^a,b^	Population: Patients with EE
Complete symptom resolution (CSR) at 1 week	117 per 1000	151 per 1000 (98 to 232)	RR 1.29 (0.84 to 1.99)	546 (2 RCTs)	⨁◯◯◯ Very low ^a,b^	Population: Patients with EE
Complete symptom resolution (CSR) at 8 weeks	326 per 1000	359 per 1000 (290 to 440)	RR 1.10 (0.89 to 1.35)	548 (2 RCTs)	⨁◯◯◯ Very low ^a,b^	Population: Patients with EE
24 h-symptom-free days at 1 week	The mean 24 h-symptom-free days at 1 week was 37.74 days	MD 2.67 days higher (2.76 lower to 8.1 higher)	-	546 (2 RCTs)	⨁◯◯◯ Very low ^a,b,c,d^	Population: Patients with EE
24 h-symptom-free days at 8 weeks	The mean 24 h-symptom-free days at 8 weeks was 66.83 days	MD 0.08 days higher (6.52 lower to 6.67 higher)	-	546 (2 RCTs)	⨁◯◯◯ Very low ^a,b,c,d^	Population: Patients with EE
4-week GERD-HRQL	The mean 4-week GERD-HRQL was 2.61	MD 0.48 higher (0.21 lower to 1.17 higher)	-	532 (2 RCTs)	⨁◯◯◯ Very low ^a,b^	Population: Patients with EE
8-week GERD-HRQL	The mean 8-week GERD-HRQL was 2.56	MD 0.37 higher (0.31 lower to 1.05 higher)	-	543 (2 RCTs)	⨁◯◯◯ Very low ^a,b^	Population: Patients with EE
Treatment-emergent adverse event (TEAEs)	339 per 1000	339 per 1000 (281 to 407)	RR 1.00 (0.83 to 1.20)	740 (3 RCTs)	⨁◯◯◯ Very low ^a,b^	Population: Patients with ARDs
Adverse drug reactions (ADRs)	117 per 1000	122 per 1000 (84 to 179)	RR 1.05 (0.72 to 1.54)	740 (3 RCTs)	⨁◯◯◯ Very low ^a,b^	Population: Patients with ARDs
Headache	14 per 1000	17 per 1000 (5 to 62)	RR 1.24 (0.34 to 4.57)	590 (2 RCTs)	⨁◯◯◯ Very low ^a,b,d^	Population: Patients with EE
Abdominal pain	17 per 1000	3 per 1000 (0 to 27)	RR 0.18 (0.02 to 1.56)	590 (2 RCTs)	⨁◯◯◯ Very low^a,b,d^	Population: Patients with EE
Dizziness	17 per 1000	16 per 1000 (3 to 90)	RR 0.96 (0.17 to 5.30)	590 (2 RCTs)	⨁◯◯◯ Very low ^a,b,c,d^	Population: Patients with EE
Nauseas	14 per 1000	7 per 1000 (1 to 37)	RR 0.50 (0.09 to 2.69)	590 (2 RCTs)	⨁◯◯◯ Very low ^a,b,d^	Population: Patients with EE
Diarrhea	20 per 1000	12 per 1000 (1 to 203)	RR 0.60 (0.04 to 9.94)	590 (2 RCTs)	⨁◯◯◯ Very low ^a,b,d,e^	Population: Patients with EE

The risk in the intervention group, together with its 95% confidence interval, is derived from the presumed risk in the comparison group and the relative effect of the intervention, including its 95% confidence interval. * Anticipated absolute effect represents the difference between the risk in the intervention group and the risk in the control group. ⨁◯◯◯ Very low certainty evidence: We have very low confidence in the effect estimate: the true effect is likely to be substantially different from the estimated effect. ARD: Acid reflux-related disorder; EE: Erosive Esophagitis; CI: confidence interval; MD: mean difference; RR: risk ratio. Explanations: ^a^. Most studies have a high risk of bias. ^b^. Pooled effect not statistically significant. ^c^. I^2^: 30–60%. ^d^. Very wide confidence interval. ^e^. I^2^ > 60%.

## Data Availability

The original contributions presented in this study are included in the article. Further inquiries can be directed to the corresponding author.

## Supplementary Materials

The following supporting information can be downloaded at: https://www.mdpi.com/article/10.3390/jcm15041434/s1, Figure S1: Forest plot of pooled estimates for complete symptom resolution (CSR) at 8 weeks; Figure S2: Forest plot of pooled 24 h symptom-free days at 1 week; Figure S3: Forest plot of pooled 24 h symptom-free days at 8 weeks; Figure S4: Forest plot of pooled 4-week GERD-HRQL; Figure S5: Forest plot of pooled 8-week GERD-HRQL; Figure S6: Forest plot of pooled estimates for treatment-emergent adverse event (TEAEs); Figure S7: Forest plot of pooled estimates for adverse drug reactions (ADRs); Figure S8: Forest plot of pooled estimates for headache; Figure S9: Forest plot of pooled estimates for abdominal pain; Figure S10: Forest plot of pooled estimates for dizziness; Figure S11: Forest plot of pooled estimates for nauseas; Figure S12: Forest plot of pooled estimates for diarrhea; Table S1: PRISMA 2020 Checklist; Table S2: Search strategy per searched electronic databases; Table S3: Excluded studies and reasons for exclusion of studies from the systematic review.
